# Supplementary material for: Waldenström’s Macroglobulinemia in a Normoproteinemic Dog with Atypical Bimorphic Plasmacytoid Differentiation and Monoclonal Gammopathy
Source: Vet Sci. 2023 May 16;10(5):355. doi: 10.3390/vetsci10050355 (PMC10222389; doi:10.3390/vetsci10050355)

Agarose gel serum protein electrophoresis from a Small Munsterlander dog with Waldenström's macroglobulinemia and other patients (Hydrasys; SEBIA, France).

Controls are on the far left: Normal control (lane 1, "N") and Hypergammaglobulinemia control (lane 2, "H").

The Small Munsterlander dog with Waldenström's macroglobulinemia is represented on the lane 3 (identification number "5806") and shows one intense and restricted band corresponding to the $\alpha_2$ globulin fraction.

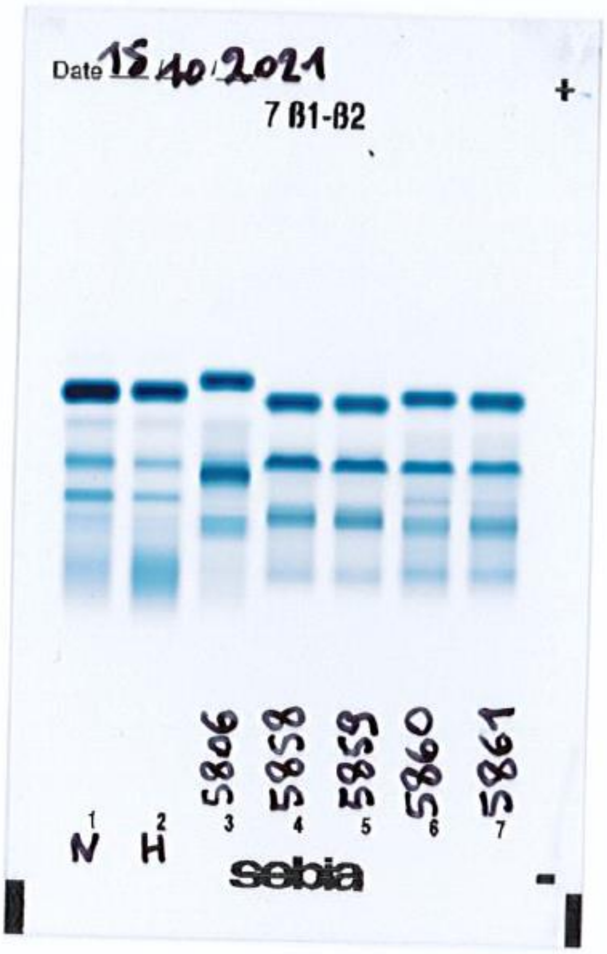

Supplement: Supplementary file 1 [file vetsci-10-00355-s001.zip › Figure S1.pdf]
