# Supplementary material for: Waldenström’s Macroglobulinemia in a Normoproteinemic Dog with Atypical Bimorphic Plasmacytoid Differentiation and Monoclonal Gammopathy
Source: Vet Sci. 2023 May 16;10(5):355. doi: 10.3390/vetsci10050355 (PMC10222389; doi:10.3390/vetsci10050355)

Serum protein immunofixation electrophoresis from a Small Munsterlander dog with Waldenström’s macroglobulinemia.

ELP: no antisera / nonspecific staining
2: heavy chains (G, A, M) polyclonal antisera
3: anti-IgG4 canine antisera
4: anti-IgA canine antisera
5: anti-IgM canine antisera
6: anti-total bound light chains canine antisera
7: anti-free and bound kappa light chains antisera
8: anti-free and bound lambda light chains antisera

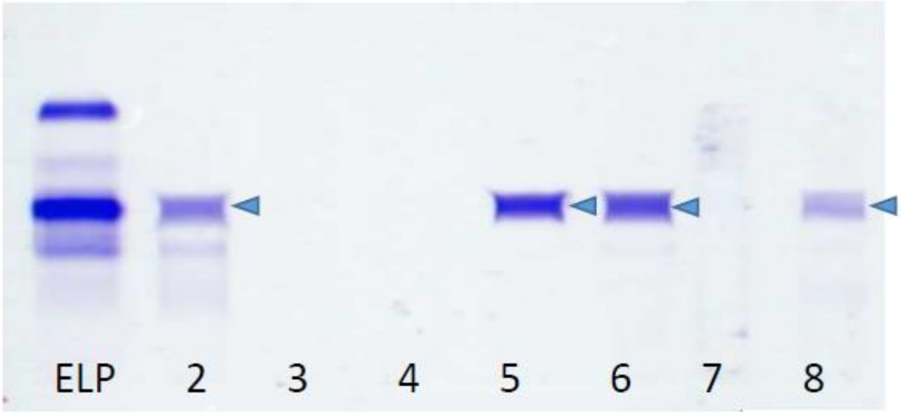

Supplement: Supplementary file 1 [file vetsci-10-00355-s001.zip › Figure S2.pdf]
