# Supplementary material for: Waldenström’s Macroglobulinemia in a Normoproteinemic Dog with Atypical Bimorphic Plasmacytoid Differentiation and Monoclonal Gammopathy
Source: Vet Sci. 2023 May 16;10(5):355. doi: 10.3390/vetsci10050355 (PMC10222389; doi:10.3390/vetsci10050355)

Urine protein electrophoresis (sodium dodecyl sulfate agarose gel electrophoresis, Hydrasys, SEBIA, France).) from a Small Munsterlander dog with Waldenström's macroglobulinemia and other patients.

Molecular weights are on the far right (lane 5, "MM"), with from top to bottom 14 kDA, 26 kDA, 66 kDA (albumin) and 150 kDA.

The Small Munsterlander dog with Waldenström's macroglobulinemia is on the far left (lane 1, identification number "5806") and shows one restricted band between 26 and 66 kDa.

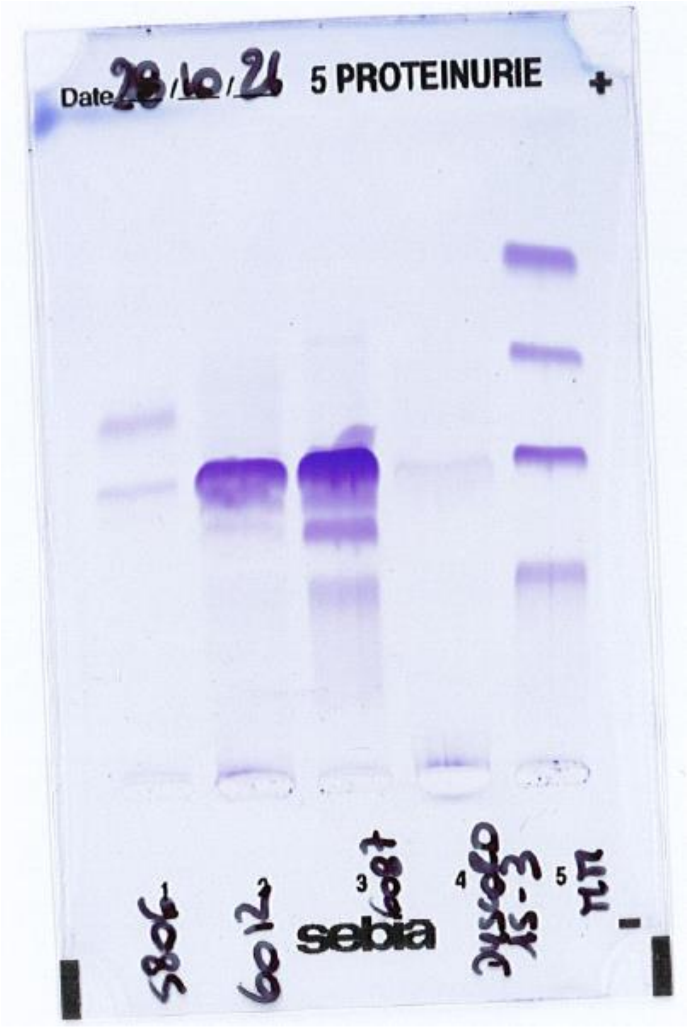

Supplement: Supplementary file 1 [file vetsci-10-00355-s001.zip › Figure S3.pdf]
