# Supplementary material for: Waldenström’s Macroglobulinemia in a Normoproteinemic Dog with Atypical Bimorphic Plasmacytoid Differentiation and Monoclonal Gammopathy
Source: Vet Sci. 2023 May 16;10(5):355. doi: 10.3390/vetsci10050355 (PMC10222389; doi:10.3390/vetsci10050355)
Supplement: Supplementary file 1 [file vetsci-10-00355-s001.zip › Supplementary captures.pdf]

Figure S1

Agarose gel serum protein electrophoresis from a Small Munsterlander dog with Waldenström's macroglobulinemia and other patients (Hydrasys; SEBIA, France).

Controls are on the far left: Normal control (lane 1, "N") and Hypergammaglobulinemia control (lane 2, "H").

The Small Munsterlander dog with Waldenström's macroglobulinemia is represented on the lane 3 (identification number "5806") and shows one intense and restricted band corresponding to the α_2 globulin fraction.

Figure S2

Serum protein immunofixation electrophoresis from a Small Munsterlander dog with Waldenström's macroglobulinemia.

ELP: no antisera / nonspecific staining
2: heavy chains (G, A, M) polyclonal antisera
3: anti-IgG4 canine antisera
4: anti-IgA canine antisera
5: anti-IgM canine antisera
6: anti-total bound light chains canine antisera
7: anti-free and bound kappa light chains antisera
8: anti-free and bound lambda light chains antisera:

Figure S3

Urine protein electrophoresis (sodium dodecyl sulfate agarose gel electrophoresis, Hydrasys, SEBIA, France).) from a Small Munsterlander dog with Waldenström's macroglobulinemia and other patients.

Molecular weights are on the far right (lane 5, "MM"), with from top to bottom 14 kDA, 26 kDA, 66 kDA (albumin) and 150 kDA.

The Small Munsterlander dog with Waldenström's macroglobulinemia is on the far left (lane 1, identification number "5806") and shows one restricted band between 26 and 66 kDa.

Figure S4

Urine protein immunofixation electrophoresis from a Small Munsterlander dog with Waldenström's macroglobulinemia.

ELP: no antisera / nonspecific staining
2: heavy chains (G, A, M) polyclonal antisera
3: anti-free and bound kappa light chains antisera
4: anti-free and bound lambda light chains antisera
5: anti-total bound light chains canine antisera
6: anti-IgM canine antisera
